# A Logistic Regression Model for Predicting Osteoporosis Using Alveolar Bone Mineral Density Measured on Intraoral Radiographs Combined with Panoramic Mandibular Cortical Index

**DOI:** 10.3390/jcm14207198

**Published:** 2025-10-13

**Authors:** Satoshi Okubo, Satoru Miyabe, Yoshitaka Kise, Tsutomu Kuwada, Akiko Hirukawa, Kenichi Gotoh, Akitoshi Katsumata, Naoki Shibata, Takahiko Morotomi, Soma Okada, Satoshi Watanabe, Toru Nagao, Eiichiro Ariji, Mitsuo Goto

**Affiliations:** 1 Department of Oral and Maxillofacial Surgery, School of Dentistry, Aichi Gakuin University, Nagoya 464-8651, Japan; 2 Department of Oral and Maxillofacial Radiology, School of Dentistry, Aichi Gakuin University, Nagoya 464-8651, Japan; 3 Division of Radiological Technology, Aichi Gakuin University Dental Hospital, Nagoya 464-8651, Japan; 4 Department of Oral Radiology, Asahi University School of Dentistry, Mizuho 501-0296, Japan; 5 Department of Endodontics, School of Dentistry, Aichi Gakuin University, Nagoya 464-8651, Japanmorotomi@dpc.agu.ac.jp (T.M.)

**Keywords:** alveolar process, bone density, radiography, dental, panoramic, osteoporosis

## Abstract

**Background**: Osteoporosis screening in dental practice is challenging because dual-energy X-ray absorptiometry is not easily applicable to jaw bones. Objective: This study aimed to evaluate the diagnostic performance of a logistic regression model combining intraoral bone mineral density (BMD) using DentalSCOPE with the panoramic mandibular cortical index (MCI) for osteoporosis screening. **Methods**: Among 104 patients included in the study, 83 who underwent both intraoral and panoramic radiography were retrospectively selected as a training cohort to develop a logistic regression model for osteoporosis prediction. The mean age was 52.4 years, and 65.1% were female. Intraoral radiographs were analyzed using DentalSCOPE^®^ (Media Co., Tokyo, Japan) to determine BMD in the alveolar region (al-BMD). On panoramic radiographs, experienced radiologists determined the MCI. An additional 21 patients (mean age 63.1 years; 81.0% female) were prospectively enrolled as an external validation cohort. The trained model was applied to both the training (internal) and external cohorts to evaluate its diagnostic performance, which was compared with that of intraoral or panoramic radiography, using receiver operating characteristic (ROC) analysis. **Results**: In the training cohort, areas under the ROC curve (AUCs) of al-BMD and MCI were 0.74 and 0.82, respectively, while the combined model showed improved performance with an AUC of 0.88. In the external validation cohort, the AUCs were 0.92 and 0.97 for al-BMD and MCI, respectively. The performance of the combined model improved with an area under the AUC of 1.00. **Conclusions**: DentalSCOPE-based al-BMD, particularly when combined with panoramic MCI, offers a reliable and practical approach for opportunistic osteoporosis screening in dental care.

## 1. Introduction

Bone mineral density (BMD) is one of the major determinants of bone strength [1,2], and its measurement plays a central role in diagnosing osteoporosis, predicting fracture risk, and monitoring therapeutic effects [3]. According to the National Institutes of Health Consensus Conference, approximately 70% of bone strength can be attributed to BMD, while the remaining 30% is related to bone quality, including microarchitecture and turnover [4]. Currently, dual-energy X-ray absorptiometry (DXA) is considered the gold standard for measuring BMD [5]. According to current guidelines, the diagnosis of osteoporosis is based on central DXA measurements at the lumbar spine, total hip, and femoral neck [6]. In the context of an aging society, the early detection of osteoporosis remains a critical clinical and public health challenge [1]. Although central DXA is the gold standard for osteoporosis diagnosis, it is not routinely performed in dental settings. Therefore, dental imaging modalities may provide an opportunity to screen patients who could benefit from further referral for DXA. Therefore, alternative screening approaches using routinely acquired dental radiographs have emerged as a promising tool [4,5,7]. Although DXA of the jawbone is not included in current diagnostic guidelines—which recommend central DXA at the lumbar spine and hip—it is not feasible to perform central DXA during routine dental visits. Therefore, indirect indicators visible on dental radiographs (such as mandibular cortical morphology or intraoral BMD) may serve as useful opportunistic screening tools to identify individuals who should be referred for further DXA evaluation.

In dentistry, BMD evaluation has been explored using various microdensitometry techniques applied to intraoral radiographs [8,9]. These techniques have been utilized not only for osteoporosis screening [10] but also for assessing alveolar bone conditions relevant for periodontal, endodontic, and surgical procedures [11,12]. Previous studies have demonstrated that bone characteristics visible on panoramic radiographs, such as the mandibular cortical width, mandibular cortical index (MCI), and trabecular patterns, correlate with systemic BMD and fracture risk [11,13,14]. Therefore, panoramic radiography has been suggested as a potential screening tool for osteoporosis [15,16,17].

Recent advances in digital imaging technology have enabled the development of computer-aided BMD measurement systems for intraoral radiography. One such system is DentalSCOPE^®^ (Media Co., Tokyo, Japan), which combines conventional microdensitometry techniques with digital image analysis [18]. This system uses calcium carbonate reference objects with defined BMD values and allows for the quantitative assessment of alveolar BMD (al-BMD) in arbitrary regions on intraoral images. DentalSCOPE offers an objective alternative to the traditional qualitative evaluation of trabecular patterns, which is highly dependent on the examiner’s interpretation [19].

The clinical significance of al-BMD is particularly evident in patients receiving antiresorptive agents, such as bisphosphonates or denosumab [20], which are associated with medication-related osteonecrosis of the jaw (MRONJ). Although reduced al-BMD and local infections are believed to contribute to MRONJ, it is considered a potential, rather than inevitable, side effect—especially in osteoporotic patients with otherwise good general health. Furthermore, MRONJ may also occur in oncologic patients receiving high-dose antiresorptive therapy or other risk-enhancing treatments. However, conclusive evidence on the etiology remains lacking.

With the global increase in the aging population, the prevalence of osteoporosis and its associated morbidity continues to rise [21]. Early identification of individuals at risk is crucial for initiating timely interventions that can prevent fractures and reduce healthcare costs. In many developed countries, individuals over the age of 65 regularly visit dental clinics for routine care. This offers a unique opportunity to implement opportunistic screening for osteoporosis in a noninvasive and cost-effective manner. The integration of dental imaging into systemic health assessments is consistent with the concept of oral systemic healthcare, which emphasizes the interconnection between oral and general health. Thus, utilizing routine dental radiographs for osteoporosis screening may serve as a practical and scalable approach for public health strategies.

In this study, we aimed to evaluate the potential of a logistic regression model that combines al-BMD provided by DentalSCOPE^®^ and MCI from panoramic images for detecting osteoporosis, compared to assessments based solely on either procedure.

## 2. Materials and Methods

### 2.1. Study Participants

**Figure 1:**
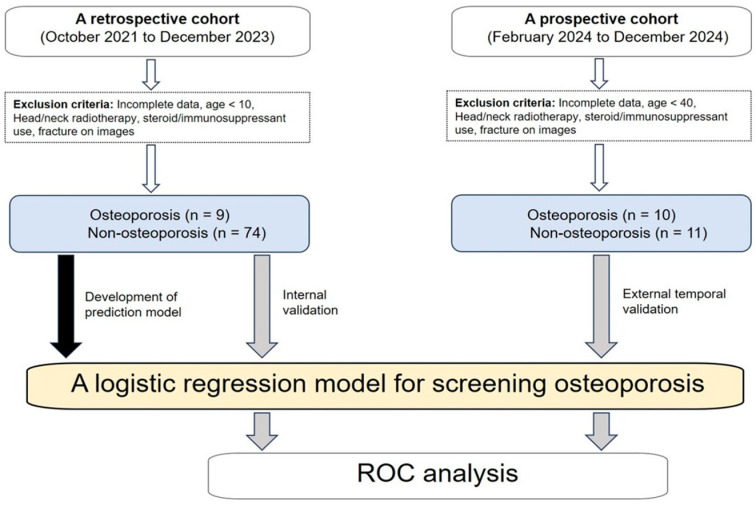
Study flow diagram.

This study comprised both retrospective and prospective observational investigations, corresponding to internal and external temporal cohorts, respectively. First, a retrospective cohort was collected from October 2021 to December 2023. Participants were included if they underwent both intraoral and panoramic radiography and met the inclusion criteria. The exclusion criteria were: (1) incomplete clinical or imaging data, (2) age < 10 years, (3) prior head and neck radiotherapy, (4) systemic steroid or immunosuppressive therapy, and (5) evidence of jaw fracture on radiographs. The presence or absence of osteoporosis was determined based on DXA criteria. In both cohorts, patients who self-reported a prior diagnosis of osteoporosis were classified into the osteoporosis group. These diagnoses were made by primary care physicians based on DXA, following national guidelines. Although the DXA results themselves were not available, the diagnosis preceded the dental imaging performed at our institution. The time interval between DXA and dental imaging varied among individuals and was not controlled in this study. Second, an external temporal validation cohort was prospectively enrolled at the same institution between February and December 2024 at the same institution. The same inclusion and exclusion criteria were applied. Although the exclusion and diagnostic criteria for osteoporosis were identical to those used for the training cohort, this cohort was limited to individuals aged ≥ 40 years to reduce the potential effects of age-related differences. Of these, 10 patients were diagnosed with osteoporosis, while the remaining 11 were classified as non-osteoporotic (Figure 1).

### 2.2. Intraoral Radiography and Bone Mineral Density Measurement

**Figure 2:**
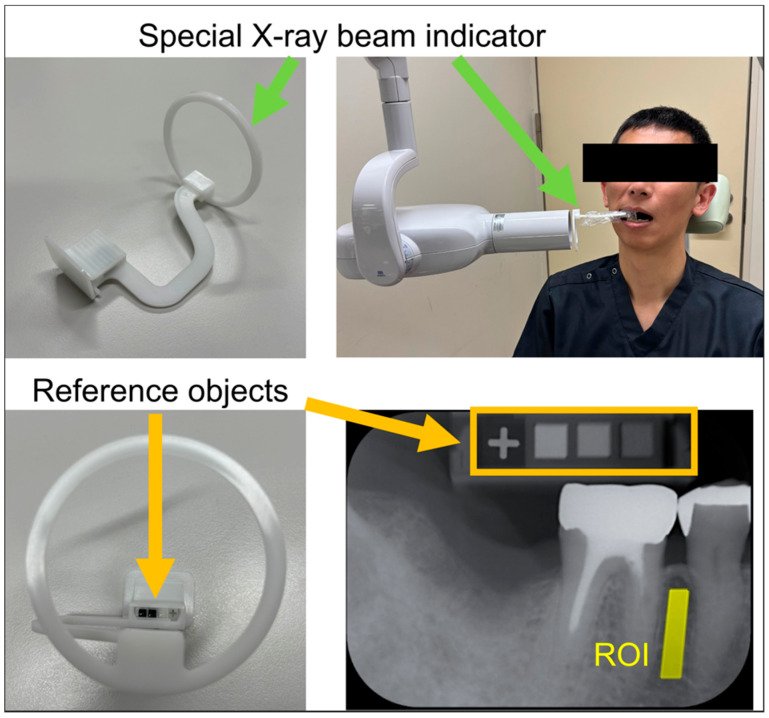
DentalSCOPE^®^ imaging system and al-BMD measurement.

al-BMD was measured using intraoral radiographs analyzed with the DentalSCOPE system. This system comprised a custom-made intraoral X-ray position-indicating device and dedicated software for BMD calculation (Figure 2). The indicating device included embedded reference objects composed of calcium carbonate at concentrations of 20%, 60%, and 100%, which allowed the software to calibrate the grayscale values accurately. The software automatically recognized these reference densities and calculated the BMD value of an arbitrary region of interest (ROI) within the alveolar bone. Intraoral radiographs were obtained using an intraoral X-ray unit (ALURA-TS, Asahi Roentgen Co., Kyoto, Japan) equipped with dedicated indicators. Images were captured on imaging plates, which were read using the Arcana Mira system (CROSS TECH Co., Tokyo, Japan) in standard mode (Figure 2). Exposure parameters were set to 60 kV and 6 mA, with exposure times ranging from 0.08 to 0.16 s. The acquired images were then imported into the DentalSCOPE software. ROIs were manually placed in the alveolar bone, carefully to avoid the roots and cortical bone (Figure 2). The size of each ROI was approximately 20 cm^2^, ranging between 18 and 22 cm^2^.

The system includes a custom-designed X-ray beam indicator with embedded calcium carbonate reference objects (top left and bottom left). Intraoral radiographs were obtained with standardized positioning (top right), and al-BMD was calculated in a defined ROI near the alveolar bone (bottom right) using digital microdensitometry. Abbreviations: al-BMD: alveolar bone mineral density

### 2.3. Panoramic Mandibular Cortical Index Evaluation

All panoramic images were independently assessed by two board-certified oral and maxillofacial radiologists, focusing on the inferior border of the mandible, based on the MCI classification [22]. In cases of disagreement, a consensus was reached through discussion.

Class 1: The endosteal margin is even and clearly defined.

Class 2: Semilunar defects or cortical thinning are observed.

Class 3: The cortex is markedly eroded or porous, with heavy endosteal residue.

### 2.4. Development and Evaluation of a Logistic Regression Model

An osteoporosis prediction model was developed from the training cohort using a logistic regression analysis. Variables that showed statistically significant differences (*p* < 0.05) in univariate analyses (Table 1)—including age, sex, MMS (mandibular morphology score), and intraoral BMD measured via DentalSCOPE—were included as explanatory variables. The dependent variable was the presence or absence of osteoporosis, as determined by DXA criteria. No regularization or cross-validation techniques were applied in this initial exploratory model. The model was constructed using the entire training dataset to maximize statistical power. The cutoff threshold for classifying a subject as osteoporotic was determined based on the Youden Index derived from the ROC curve, defined as the point maximizing (sensitivity + specificity − 1). These methodological details have now been explicitly described to improve clarity and reproducibility. Variables that showed statistically significant differences (*p* < 0.05) in univariate analyses (Table 1) were included as explanatory variables.

For internal validation, the developed model was applied to an internal (training) cohort, and its performance was assessed using receiver operating characteristic (ROC) analysis by calculating the area under the ROC curve (AUC). The optimal cutoff value was determined from the ROC curve using the Yoden index, defined as the maximum value of sensitivity + specificity − 1. Based on this cutoff, the sensitivity, specificity, and accuracy of the model were calculated. Subsequently, the model was applied to a prospectively collected external validation cohort, and its predictive performance was assessed using the same methods used for the internal validation. For comparison, the resulting al-BMD or MCI was also used solely as a predictor, and its performance was assessed using the same methods as those used in the regression model.

### 2.5. Ethical Approval

This study was approved by our university’s ethics committee (approval no. 631, approved in 2021) and conducted in accordance with the Declaration of Helsinki.

### 2.6. Statistical Analysis

Differences in age and al-BMD between the osteoporosis and non-osteoporosis groups were evaluated using the *t*-test, while differences in the proportions of other indices were assessed using the chi-square test. The difference in AUCs among the prediction methods was also evaluated using a Chi-square test. All statistical analyses were performed using JMP Pro, software version 18.2.0 (SAS Institute Inc., Cary, NC, USA), with a significance level set at *p* < 0.05.

## 3. Results

A total of 83 patients were included in the training (retrospective) cohort and 21 in the external temporal validation (prospective) cohort, following the application of inclusion and exclusion criteria.

### 3.1. Internal Validation Using the Training Cohort

**Figure 3:**
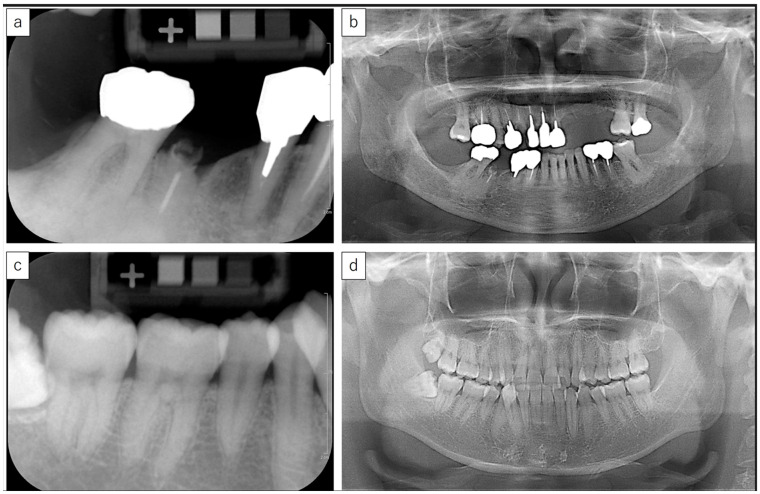
Representative intraoral and panoramic radiographs of osteoporotic and non-osteoporotic cases. Representative intraoral radiograph and panoramic images of an osteoporotic case (**a**,**b**) and non-osteoporotic case (**c**,**d**). Note the sparse trabecular structure and cortical erosion in (**a**,**b**) compared with the denser trabecular pattern and intact cortex in (**c**,**d**).

**Figure 4:**
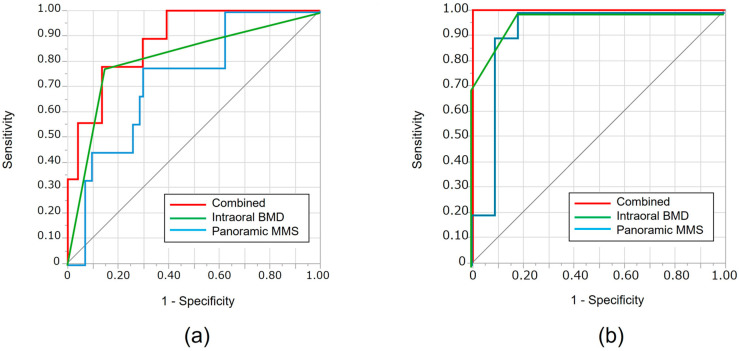
ROC curves for predicting osteoporosis using al-BMD alone, panoramic MCI alone, and the combined regression model. (**a**) Internal (training) validation; (**b**) External validation. The combined regression model showed superior diagnostic performance compared to each modality alone. Abbreviations: MCI: mandibular cortical index; al-BMD: alveolar bone mineral density.

Of the 83 patients included in the training cohort, 9 were diagnosed with osteoporosis based on prior DXA evaluations, while the remaining 74 were classified as non-osteoporotic. Representative intraoral and panoramic radiographic findings of patients with and without osteoporosis are presented in Figure 3. Based on the characteristics of patients in this cohort (Table 1), the univariable analyses revealed significant differences in age, al-BMD, and MCI between those with and without osteoporosis. A multivariate logistic regression model was then developed based on these variables. Consequently, al-BMD and MCI were found to be significant predictors in the model. The AUC for this combined model was 0.88, which was higher than the AUCs for al-BMD (0.74) or MCI (0.82) when used individually; however, the difference was not statistically significant (Table 2, Table 3, and Figure 4).

### 3.2. External Validation Using the Temporal Cohort

The combined regression model demonstrated perfect performance, with an AUC of 1.00. This indicates a slight, but not statistically significant, improvement compared to the AUCs of al-BMD (0.92) and MCI (0.97) when used individually (Table 2, Figure 4).

## 4. Discussion

In this study, al-BMD in the internal cohort demonstrated relatively modest performance, with an AUC and accuracy of 0.74. This level is comparable to values reported in previous panoramic-based osteoporosis screening studies [6,7], supporting the feasibility of using al-BMD measurements in dental settings. When comparing al-BMD and MCI as individual predictors, MCI showed superior performance in both the internal and external validations. This may be partly due to the small number of patients with osteoporosis in the training cohort, which may have limited the robustness of the al-BMD model. Moreover, the panoramic assessments were performed by oral and maxillofacial radiologists who were highly experienced in MCI evaluation, possibly contributing to its better classification performance. Furthermore, age was significantly associated with osteoporosis status in both cohorts (Table 1), underscoring its role as a confounder in age-related BMD decline. Although age was excluded from the final regression model to avoid overfitting, this distributional imbalance should be considered when interpreting screening accuracy.

Notably, the external validation cohort (*n* = 21) demonstrated an outstanding diagnostic performance, with an AUC of 1.00. Although this finding may have been partially influenced by the limited sample size, it nonetheless provides compelling evidence for the robustness and generalizability of the combined model. However, the perfect performance should be interpreted with caution, as it may indicate a risk of overfitting due to the relatively small sample size. Further external validation in larger and more diverse populations is warranted. These findings are consistent with previous research highlighting the value of mandibular radiomorphometric indices and digital densitometry in identifying osteoporosis [9,10,11].

From a clinical perspective, intraoral radiographs are acquired more commonly than panoramic images during routine dental practice, which enhances the accessibility of al-BMD assessments [12,13]. Moreover, combining the MCI parameters provided complementary diagnostic information, with AUCs of 0.88 and 1.00 for the internal and external validations, respectively. This integrated imaging strategy may allow dentists to contribute more proactively to systemic disease screening, in line with the concept of oral-systemic healthcare [23]. Because intraoral and panoramic radiographs are already commonly acquired during routine dental examinations, this method of osteoporosis screening could be implemented opportunistically—without additional imaging or radiation exposure—thereby enhancing early detection in everyday clinical practice. For example, mandibular cortical parameters derived from panoramic radiographs have been validated against spinal osteoporosis [14,15], and recent studies have reported the potential of artificial intelligence-assisted analysis of panoramic images for osteoporosis detection [17]. Currently, no international guidelines recommend the use of intraoral or panoramic radiographs as standalone tools for osteoporosis screening. However, several studies—including the present study—have reported diagnostic performance with AUC values exceeding 0.80, supporting their potential as opportunistic screening tools. In Japan, some university hospitals and local institutions have begun to implement such assessments in clinical settings, particularly for aging patients who undergo dental imaging for other purposes. Therefore, while these tools cannot yet replace DXA, they may serve as practical adjuncts to identify at-risk individuals who require further evaluation.

In addition to osteoporosis screening [17], al-BMD measurements using DentalSCOPE have broader clinical promises [24]. In periodontal treatment, serial BMD assessments may serve as objective markers of alveolar bone regeneration [18]. In implant dentistry, BMD values can assist in predicting primary implant stability and optimizing surgical planning [19]. In orthodontics, regional BMD may help in evaluating bone responsiveness to mechanical loading. Moreover, site-specific BMD analysis may help assess the risk of MRONJ, especially in patients receiving antiresorptive therapy [20,25].

However, this study has certain limitations. First, BMD measurements were restricted to localized alveolar bone regions, which may not fully reflect systemic bone status. Therefore, future studies should include the mandibular ramus and condyle, where the effects of focal dental stress and inflammation may be minimized. Second, the external validation cohort was relatively small, which potentially affected the diagnostic metrics. Therefore, external validation across different institutions is needed. Third, MCI remains qualitative and examiner-dependent. Future developments, such as deep learning-based analysis, may improve objectivity and reproducibility, even when applied by inexperienced observers [26,27,28]. Additionally, the study population may have included a heterogeneous mix of osteoporosis, osteopenia, and normal bone mineral density based on prior DXA diagnoses. This heterogeneity could have influenced the performance of the predictive models and should be addressed in future studies with more granular categorization and larger sample sizes. As this was a pilot study with a relatively small sample size, further research involving larger and more diverse populations is needed. Future investigations should particularly focus on high-risk subgroups, such as postmenopausal females and older adults, to assess the broader applicability of these screening tools in clinical practice.

## 5. Conclusions

This study’s findings support the combined use of al-BMD measurements obtained with DentalSCOPE and MCI values from panoramic radiography as a practical and accurate method for osteoporosis screening in dental clinics. Given its accessibility, objectivity, and high diagnostic performance, this approach holds promise for incorporation into opportunistic public health strategies for early detection of osteoporosis.

## Figures and Tables

**Table 1:** Subjects’ characteristics.

Characteristics		Training (Internal Validation) Cohort (*n* = 83)			External (Temporal) Validation Cohort (*n* = 21)		
		Osteoporosis (*n* = 9)	Non-Osteoporosis (*n* = 74)	*p* Value	Osteoporosis (*n* = 10)	Non-Osteoporosis (*n* = 11)	*p* Value
Sex	Female	8	46	0.150	9	8	0.59
	Male	1	28		1	3	
Age (years)	Mean ± SD	75.4 ± 10.8	51.9 ± 22.4	0.010	76.1 ± 8.3	54.2 ± 11.1	0.022
al- BMD (mg/cm^2^)	Mean ± SD	858.5 ± 179.4	1016.6 ± 232.3	0.032	773.2 ± 192.7	1204.6 ± 295.6	0.019
MCI	class 1 and 2	2	63	0.003	3	11	<0.001
	class 3	7	11		7	0	
History of steroid use	Present	1	2	0.29	0	0	<0.001
	Absent	8	72		10	11	
Diabetes	Present	0	4	1.00	1	0	0.48
	Absent	9	70		9	11	
History of malignant tumors	Present	1	3	0.37	0	2	0.48
	Absent	8	71		10	9	
Thyroid disease	Present	0	6	1.00	1	3	0.59
(Graves’ disease, hypothyroidism, chronic thyroiditis)	Absent	9	68		9	8	

BMD, bone mineral density measured on intraoral radiograph; MCI, mandibular cortical index on panoramic radiograph; SD, standard deviation. Unless otherwise specified, variables are presented as number of patients.

**Table 2:** Diagnostic performance of al-BMD, panoramic MCI, and combined models in the training and temporal validation cohorts.

	Training (Internal Validation) Cohort (*n* = 83)			External (Temporal) Validation Cohort (*n* = 21)		
	al-BMD Alone	MCI Alone	Combined Model	al-BMD Alone	MCI Alone	Combined Model
Cutoff value	884.8	Class 2	0.23	1067.1	Class 2	0.13
AUC	0.74	0.82	0.88	0.92	0.97	1.00
Sensitivity	0.78	0.78	0.87	0.82	1.00	1.00
Specificity	0.70	0.85	0.78	1.00	0.82	1.00
Accuracy	0.71	0.86	0.86	0.91	0.91	1.00

al-BMD: alveolar bone mineral density, MCI: mandibular cortical index.

**Table 3:** Multivariate logistic regression results for predictors of osteoporosis (training cohort).

	Multivariate Analysis	
Variable	OR (95% CI)	*p*-Value
Age	0.033 (5.078 × 10^−5^–29.9)	0.21
al-BMD values from intraoral radiographs	10.3 (3.69–15.8)	0.014
MCI from panoramic radiographs	0.10 (0.0064–9.81)	0.033

Note. BMD: Bone Mineral Density values; CI: confidence interval; MCI: mandibular cortical index; OR: odds ratio.

## Data Availability

The data presented in this study are available upon request from the corresponding author. The names and exact data of the study participants are not available owing to patient confidentiality and privacy policies.

## Abbreviations

The following abbreviations are used in this manuscript:

al-BMD	Alveolar Bone Mineral Density
AUC	Area Under the Curve
BMD	Bone Mineral Density
DXA	Dual-energy X-ray Absorptiometry
MCI	Mandibular Cortical Index
MRONJ	Medication-Related Osteonecrosis of the Jaw
ROC	Receiver Operating Characteristic
ROI	Region of Interest
